# Temperature-Dependent Residual Stress and Optical Properties of Asymmetric W-Doped VO_2_-Based Trilayer Thin Films

**DOI:** 10.3390/ma19081585

**Published:** 2026-04-15

**Authors:** Chuen-Lin Tien, Chun-Yu Chiang, Lung-Shun Shih, Ching-Chiun Wang, Shih-Chin Lin

**Affiliations:** 1 Department of Electrical Engineering, Feng Chia University, Taichung 40724, Taiwan; 2 Ph.D. Program of Electrical and Communications Engineering, Feng Chia University, Taichung 40724, Taiwan; hank4681898@gmail.com (C.-Y.C.); p1435176@o365.fcu.edu.tw (L.-S.S.); 3 Mechanical and Systems Research Lab, Industrial Technology Research Institute, Hsinchu 310401, Taiwan; juin0306@itri.org.tw (C.-C.W.); shihchin@itri.org.tw (S.-C.L.)

**Keywords:** W-doped vanadium dioxide, multilayer thin film, phase transition temperature, residual stress, thermal expansion coefficient

## Abstract

This study aims to reduce the phase transition temperature (PTT) of W-doped vanadium dioxide (VO_2_) multilayer thin films. We designed and fabricated two asymmetric multilayer thin film structures; namely, TiO_2_/VO_2_-5%W/ITO and ITO/VO_2_-5%W/TiO_2_. The W-doped VO_2_-based Trilayer thin films were deposited using an electron beam evaporation combined with the ion-assisted deposition (IAD) technique. An experimental study was conducted on the temperature-dependent residual stress and optical properties of the two asymmetric VO_2_-based three-layer structures. The VO_2_-based thin films were characterized using UV–Vis–NIR spectrophotometry, Fourier transform infrared spectroscopy (FTIR), Raman spectroscopy, and an improved Twyman–Green interferometer combined with fast Fourier transform (FFT) analysis for residual stress measurement. The trilayer structures incorporated a ~60 nm thick W-doped VO_2_ middle layer, which plays a critical role in modulating thermochromic behavior and residual stress evolution. The results show that both trilayer thin films demonstrated excellent optical performance in transmission spectra. Raman spectral analysis revealed a blue shift in the characteristic W-doped VO_2_ peaks, accompanied by a decrease in peak intensity as the temperature increased. Heating experiments on asymmetric W-doped VO_2_ trilayer thin films revealed that the critical transition temperature of the ITO/VO_2_-5%W/TiO_2_/B270 trilayer film structure was significantly reduced to 45 °C. This demonstrates the effectiveness of our proposed multilayer film design in improving the PTT of W-doped VO_2_ thin films. Analysis of the changes in residual stress of the trilayer thin films during heating experiments revealed that the residual stress shifted from compressive to tensile in the temperature range of 40 °C to 50 °C. The thermal expansion coefficient and biaxial modulus of the TiO_2_/VO_2_-5%W/ITO trilayer film structure were 5.37 × 10^−6^ °C^−1^ and 295.7 GPa, respectively. In addition, the thermal expansion coefficient and biaxial modulus of the ITO/VO_2_-5%W/TiO_2_ trilayer film structure were 6.65 × 10^−6^ °C^−1^ and 745.0 GPa.

## 1. Introduction

The application of smart windows in modern architecture has become increasingly widespread due to their unique functionality, which enables effective temperature regulation within buildings. When ambient temperatures rise, smart windows can reduce heat gain by limiting solar transmission, keeping interiors cool [1,2]. Vanadium dioxide (VO_2_) is a representative material exhibiting a metal–insulator transition (MIT) [3]. For example, VO_2_ film could be used as optical switching that deposited on Al doped zinc oxide (AZO) conductive glass substrates by DC reactive magnetron sputtering. The deposited VO_2_ film exhibits excellent optical switching properties with a very small phase-transition hysteresis width of 2.9 °C as well as a low PTT of about 48 °C [4]. During the phase transition from a monoclinic insulating phase at room temperature to a rutile metallic phase at higher temperatures, VO_2_ undergoes significant changes in both optical transmittance and electrical conductivity [5]. The phase transition typically occurs around 340 K (~68 °C), which remains too high for practical smart window applications [6]. In recent years, VO_2_ has attracted considerable attention due to its pronounced and reversible changes in optical and electrical properties. Lowering the PTT of VO_2_ closer to ambient conditions is critical for maximizing its ability to regulate solar energy transmission effectively. This has driven extensive research into various methods for reducing T_t_, including metal ion doping, optimization of processing parameters, and the fabrication of multilayer composite films.

To regulate the properties of VO_2_ thin films, Case [7] investigated the influence of ion beam parameters on the optoelectronic properties of VO_2_ films deposited via ion-assisted reactive evaporation. Their study showed that the refractive index and extinction coefficient decreased with increasing oxygen flow rate during ion-assisted deposition, which also effectively reduced the transition temperature. In 2021, Zhang et al. [8] studied a VO_2_ (M)–silicon dioxide (SiO_2_) composite coating fabricated from ammonium citrato-oxovanadate (IV) using a SiO_2_-assisted coating method. The results show 49.2% visible transmittance, 52.3% transmittance reduction at a 2000 nm wavelength, 12% solar energy modulation (ΔTsol), and a PTT of 56.0 °C. AFM images revealed that as the O_2_/Ar ratio increased from 2% to 5%, the grain size of the VO_2_ films also increased. Corresponding changes in optical properties and transition temperatures were observed, with the sample prepared at 2% O_2_ exhibiting a T_t_ of 46 °C. Shen and Chen [9] provided a comprehensive review and discussion on doping and hybrid approaches to modulate VO_2_ properties. They introduced electrothermal techniques for thermochromic control and demonstrated that doping VO_2_ can achieve a solar modulation efficiency (ΔT_sol_) of 34.3% and luminous transmittance (T_lum_) of 53.9%. For electrothermal applications, transparent conductive materials such as ITO, FTO, and AZO have been employed as heating layers to thermally trigger the VO_2_ phase transition in small-scale devices. The PTT should be reduced as it approaches room temperature. In contrast, luminous transmittance (T_lum_) and solar energy modulation efficiency (ΔT_sol_) must be high enough to capture adequate daylight and exhibit an energy-saving trend. Bleu and Bourquard [10] utilized pulsed laser deposition combined with rapid thermal annealing to fabricate thermochromic BN/VO_2_/BN trilayer films. While the BN buffer layer did not significantly lower the PTT, it altered the surface morphology of the VO_2_ film. This trilayer structure significantly reduced the critical temperature (T_c_) and exhibited a wide hysteresis window, along with improved optical transmittance (T_lum_). A BN layer thickness of 70 nm resulted in a T_c_ as low as 36 °C, with a large hysteresis width (~25 °C) and a T_lum_ of ~40%. However, the ΔT_sol_ remained low. A novel structure paves the way for the development of thermochromic heterostructures with tunable properties, which could be potentially valuable for smart windows and optical data storage systems [11]. Furthermore, doping with compatible elements has been shown to effectively reduce the PTT [12,13,14,15,16,17]. Owing to its exceptional phase transition characteristics, VO_2_ is widely applied in various fields, including optoelectronic switches [18], smart windows [19], thermal imaging [20], laser protection [21], and optical memory devices [22].

In addition to experimental methods, first-principles calculations [23] and atomic-level modeling have been widely used to understand defect formation, dopant introduction, and microstructure evolution in VO_2_ thin films. For example, first-principles studies have shown that heavily doped agents (such as W) can induce lattice distortion, oxygen vacancies, and local strain fields, thus significantly affecting phase transition behavior. These modeling methods provide valuable insights into the relationship between doping, defect formation, and thermochromic properties. In this study, the optical properties and residual stress of asymmetric tungsten-doped VO_2_-based trilayer thin films exhibit temperature-dependent behavior. Tungsten doping generally reduces the critical PTT and the residual stress level within the films. Increasing the tungsten content leads to reduced residual stress and increased surface roughness. At the same time, as the thin film stack becomes more metallic at higher temperatures, optical transmittance in the visible spectrum decreases. These changes are related to the VO_2_ thin film’s transition from an insulating to a metallic state at higher temperatures, which in turn affects the lattice structure, atomic spacing, and electronic band structure. To reduce the PTT of W-doped VO_2_-based thin films, we designed and fabricated two asymmetric multilayer composite films. This paper describes and compares the TiO_2_/VO_2_-5%W/ITO/B270 and ITO/VO_2_-5%W/TiO_2_/B270 multilayer film stacks. Furthermore, visible light transmittance, mid-infrared transmittance, temperature-dependent residual stress, and Raman spectroscopy were measured and analyzed. Unlike some literature studies that primarily focused on doping or monolayer optimization, this research proposes an asymmetric three-layer vanadium oxide-based thin film design strategy that combines tungsten doping and optical interference coating, achieving simultaneous control over PTT, optical transmittance, and residual stress. Furthermore, this study combines residual stress measurement (using interferometry) with thermochromic thin film characterization, thereby providing a comprehensive and in-depth understanding of the thermomechanical coupling behavior of temperature-dependent VO_2_-based multilayer thin film devices.

## 2. Materials and Methods

### 2.1. Thin Film Design and Multilayer Preparation

The temperature-dependent optical properties and residual stress behavior of thermochromic VO_2_-based multilayer composite structures were investigated experimentally. The research focuses on comparing the optical, mechanical, and electrical characteristics of these films across varying temperatures, with particular attention to changes in transmittance and mechanical properties during the metal–insulator transition (MIT). In addition, the study examines how the integration of different materials (TiO_2_ and ITO) with VO_2_-5%W single-layer films affects the overall film properties, aiming to modify and optimize performance under thermal variation. This provides both theoretical insights and experimental data to support the development of VO_2_-based materials for smart window technologies and other related applications.

The composite VO_x_ thin films were deposited using an electron beam evaporation technique combined with ion-assisted deposition (IAD) [24,25,26]. Prior to deposition, the substrate surfaces were treated using an atmospheric pressure plasma cleaner to enhance film–substrate adhesion. The effects of various parameters on the average visible light transmittance, mid-infrared transmittance, residual stress, and Raman spectrum were evaluated. The combined electron beam evaporation and IAD technique leverages the advantages of both methods. The accelerated ion beam bombardment of the film surface helps remove micro-voids and defects, thereby improving the critical thickness and crystalline quality of the films. By adjusting the energy and incidence angle of the ion beam, the residual stress in the film can be effectively controlled, minimizing the risk of stress-induced cracking or deformation. Additionally, the ion beam enhances the adhesion between the film and the substrate by increasing interfacial bonding strength [27,28,29].

Two distinct asymmetric three-layer film structures were designed using the Essential Macleod thin-film design software, comprising different stacking sequences of high-refractive-index materials: Sub/ITO/VO_2_-5%W/TiO_2_ and Sub/TiO_2_/VO_2_-5%W/ITO. Indium tin oxide (ITO), a material with a high refractive index, exhibits high transmittance in the visible spectrum while effectively reducing transmittance in the infrared region. Additionally, ITO is chemically stable [30], making it resistant to reactions under typical environmental conditions, thereby maintaining its performance over time. Titanium dioxide (TiO_2_) is another high-refractive-index material [31], known for its stable chemical properties, strong redox capabilities, and environmental safety.

This study chose a three-layer structure to simultaneously achieve multiple functional objectives: (1) the VO_2_-5%W layer provides a thermochromic color modulation layer; (2) the TiO_2_ layer enhances visible light transmittance through its anti-reflective effect; and (3) the ITO layer serves as an infrared reflective layer and a conductive buffer layer. Each layer serves a distinct physical function. This combination allows for independent adjustment of optical and thermal properties, whereas single-layer or double-layer structures cannot achieve multiple functions and significantly reduce the PTT. The thickness of each layer was determined by using Essential Macleod software version 9.0. The VO_2_-5%W layer thickness (~60 nm) was selected as a compromise between sufficient optical modulation and maintaining high visible transmittance. Thinner films (<40 nm) resulted in reduced modulation efficiency, while thicker films (>80 nm) significantly decreased luminous transmittance due to increased absorption. Similarly, the TiO_2_ (~60 nm) and ITO (185–310 nm) layers were optimized to function as anti-reflection and infrared-reflective layers, respectively. The thickness tolerances were controlled within ±5 nm using quartz crystal monitoring. Three multilayer structures were designed to optimize the optical performance in both visible (400–750 nm) and infrared (2.5–7.5 μm) spectral regions. The design target was to achieve over 70% transmittance in the visible range and below 30% transmittance in the infrared range. Furthermore, the asymmetric three-layer structure was also designed to lower the PTT, thereby enhancing application performance.

**Figure 1:**
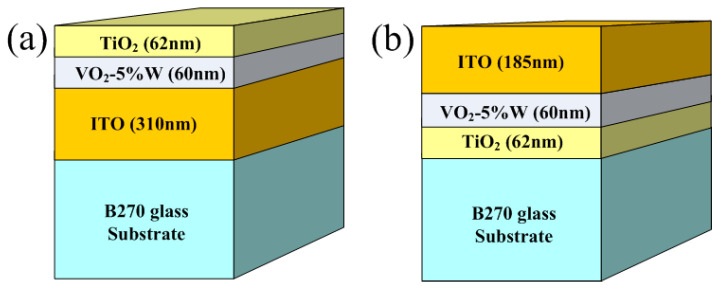
Thin film structure design (**a**) TiO_2_/VO_2_-5%W/ITO/B270 (Type I); (**b**) ITO/VO_2_-5%W/TiO_2_/B270 (Type II).

In the first structure, as illustrated in Figure 1a, ITO was deposited as the bottom layer (310 nm) to serve as an infrared shielding and buffer layer. The middle layer consists of a 60 nm thick VO_2_ film doped with 5% tungsten (VO_2_-5%W), which is responsible for modulating optical properties and reducing the PTT. The top layer is a 62 nm TiO_2_ film acting as an anti-reflection layer to enhance visible light transmittance and provide surface protection. The deposition parameters were as follows: oxygen flow rate of 80 sccm, deposition rate of 1 Å/s, and substrate temperature of 250 °C. The ion-beam-assisted deposition (IBAD) process utilized a gas flow ratio of Ar: O_2_ = 14:2. Film thickness was monitored using a quartz crystal microbalance. The second asymmetric structure, as illustrated in Figure 1b, is a reverse-stack trilayer of the first structure design, consisting of TiO_2_ (62 nm) as the first layer, VO_2_-5%W (60 nm) as the middle layer, and ITO (185 nm) as the top layer. The deposition parameters for this structure were identical to those of the first configuration. All depositions were performed using a SHOWA electron beam evaporation system integrated with ion-beam-assisted deposition (IAD) technology.

The goal of this deposition process was to optimize the physical and chemical properties of the films by combining different materials to achieve application-specific performance. The gases used in the process included high-purity oxygen (O_2_, 99.99%) as the reactive gas for oxide film formation, and high-purity argon (Ar, 99.99%) for ion bombardment during IAD to enhance film density and adhesion. Circular substrates of 30 mm diameter, B270 and H-K9L glass substrates were selected. Prior to thin film deposition, substrates were first cleaned with acetone, followed by ultrasonic cleaning to remove surface contaminants. Plasma treatment was then applied to enhance film–substrate adhesion further and improve surface uniformity. During the deposition process, VO_2_, ITO, and TiO_2_ source materials were placed in separate crucibles and evaporated by high-energy electron-beam bombardment. The resulting vapor was deposited onto the substrate to form the thin films. Film thickness and deposition rates were precisely controlled using a quartz crystal microbalance, which allowed real-time monitoring of physical thickness and rate to ensure compliance with design specifications.

### 2.2. Thin Film Measurements

**Figure 2:**
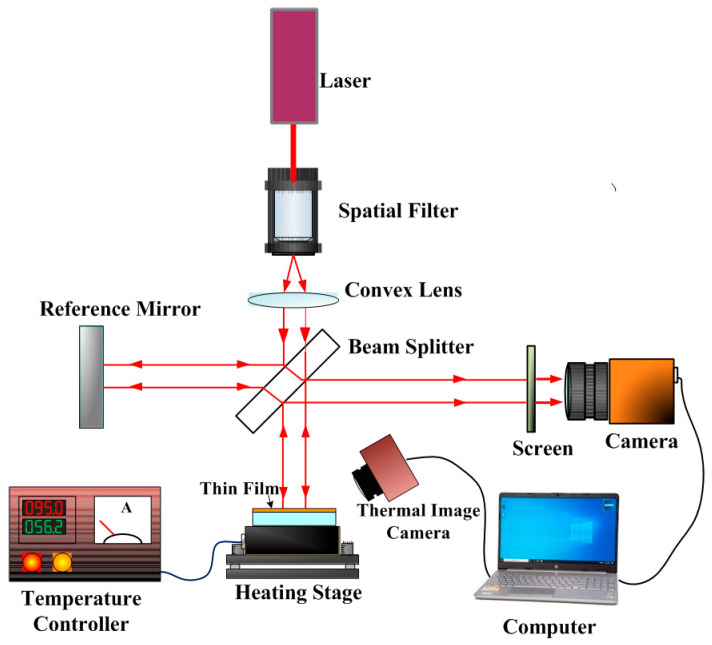
Schematic diagram of the improved Twyman–Green interferometer and resistive heating stage system.

For measuring the temperature-dependent residual stress of thin films, an improved Twyman–Green interferometer combined with Fast Fourier Transform (FFT) techniques was employed [32,33] to evaluate the residual stress of identical film materials deposited on two different glass substrates [34,35]. The system architecture is illustrated in Figure 2. By integrating this system with non-contact temperature mapping obtained via infrared thermography, the variation in residual stress at different temperatures could be determined. Based on the slope of the residual stress–temperature relationship, and by performing measurements on two different substrates, the thermal expansion coefficient and biaxial modulus of the thin films could be calculated through simultaneous equations [36]. This method leverages the high-precision surface deformation detection capability of interferometry and the data-processing advantages of FFT to provide accurate mechanical stress characterization of thin films.

The residual stress measurement setup is based on a modified Twyman–Green interferometer. A helium-neon laser with a central wavelength of 632.8 nm is used as the light source. The beam passes through a spatial filter, composed of a microscope objective and a pinhole, to remove high-frequency noise, producing a point source. A plano-convex lens then collimates this beam to form a parallel beam, which is directed onto a beam splitter. The beam splitter divides the incident beam into two paths: one is reflected onto a reference mirror, and the other is directed onto the sample surface. The two reflected beams interfere and form fringes on a projection screen, which are captured by a CCD camera. The captured interference fringes are then analyzed using a custom-developed MATLAB program version R2024b [37,38].

The analysis program processes the acquired fringe pattern by first selecting the region of interest, followed by digital filtering to eliminate noise and extract the carrier signal. FFT is then applied to extract the phase information. After phase unwrapping, the surface profile of the thin film is reconstructed. The radius of curvature of the sample is then calculated from the reconstructed surface, and the residual stress is determined using the Stoney equation [39,40]. The curvature difference between the substrate before and after thin film deposition is used in calculating residual stress. By analyzing the equal-thickness interference fringes using FFT, the phase profile of the film surface can be recovered. The film surface topography is then reconstructed, and the radius of curvature (ROC) is obtained via curve fitting. The residual stress is calculated based on the change in ROC of the substrate before and after deposition. The modified formula is expressed as:

(1) $$\sigma =\frac{E_{s}\cdot t_{s}^{2}}{6t_{f}\left(1-V_{s}\right)}\left(\frac{1}{R_{2}}-\frac{1}{R_{1}}\right)$$

In Equation (1), σ is the residual stress of the film. *R* is the ROC after thin film coating, and *R*_0_ is the ROC of the bare substrate. *E_s_* is the Young’s modulus of the substrate, *V_s_* is the Poisson’s ratio of the substrate, *t_s_* is the thickness of the substrate, and *t_f_* is the thickness of thin film.

The thermal stress (*σ_th_*) arises from the mismatch in thermal expansion between the thin film and the substrate. The thermal stress can be written as follows:

(2) $$\sigma_{th}=\left(\alpha_{s}-\alpha_{f}\right)\frac{E_{f}}{1-\nu_{f}}\left(T_{2}-T_{1}\right),$$

where α_f_ and α_s_ represent the CTEs of the substrate and thin film, respectively, and E_f_ and *ν_f_* are the Young’s modulus and Poisson’s ratio of the thin film. $\frac{E_{f}}{1-v_{f}}$ is the biaxial modulus of the thin film. *T*_1_ and *T*_2_ represent the temperature differences in the thin film before and after substrate heating. Differentiating Equation (2) with respect to the heating temperature, we obtain the following formula.

(3) $$\frac{d\sigma_{th}}{dT}=(\alpha_{s}-\alpha_{f})\frac{E_{f}}{1-\nu_{f}}.$$

Here, it is assumed that the values of *α_s_*, *α_f_*, *E_f_*, and *ν_f_* are independent of temperature. In Equation (3), both α_f_ and $\frac{E_{f}}{1-v_{f}}$, are unknown parameters, we used dual substrate method to solve two simultaneous equations, and using the slop of the stress-temperature curves to calculate *α_f_* and $\frac{E_{f}}{1-v_{f}}$, the corresponding formula is expressed as follows [16]:

(4) $$\alpha_{f}=\frac{\alpha_{1}\frac{d\sigma_{2}}{dT}-\alpha_{2}\frac{d\sigma_{1}}{dT}}{\frac{d\sigma_{2}}{dT}-\frac{d\sigma_{1}}{dT}}$$

(5) $$\frac{E_{f}}{1-\nu_{f}}=\frac{\frac{d\sigma_{2}}{dT}-\frac{d\sigma_{1}}{dT}}{\alpha_{2}-\alpha_{1}}$$

where α_1_ and α_2_ are the thermal expansion coefficients for B270 and H-K9L glass substrates, respectively; *α*_*f*_ is the CTE of the thin film; *E_f_* and *ν_f_* are the biaxial modulus and Poisson’s ratio of the thin film, respectively; and $\frac{d\sigma_{1}}{dT}$ and $\frac{d\sigma_{2}}{dT}$ are the two slope values of the stress–temperature curves of the thin film deposited on two different substrates. Table 1 shows the physical parameters of two different glass substrates.

The transmittance of the material was measured using a Shimadzu UV-2600i spectrometer, with a wavelength range of 200–900 nm. The transmission spectrum was analyzed using the envelope method [41], and the refractive index, extinction coefficient, and physical thickness of the film were obtained.

The sample’s spectral data were recorded using a visible light spectrometer (UV-2600i), and its visible light transmittance (T_lum_, λ = 400–750 nm), solar light transmittance (T_sol_, 350–2500 nm), optical modulation (ΔT_lum_), and mid-infrared transmittance (T_mir_, λ = 3000–5000 nm) were calculated. *ϕ*_lum_(λ), *ϕ*_sol_(λ), and *ϕ*_MIR_(λ) represent the weighting functions for the visible, solar, and far-infrared spectra, respectively, while T(λ) represents the transmittance at wavelength λ.

(6) $$T_{lum}=\int \emptyset_{lum}(\lambda )T(\lambda )d\lambda /\int \emptyset_{lum}(\lambda )d\lambda$$

Equation (6) is used to calculate the visible light transmittance T_lum_ by integrating the transmittance T(λ) and the visible spectrum weighting function *ϕ*_lum_(λ) over the wavelength range of 400–750 nm.

To calculate the visible light modulation rate ΔT_lum_, which is the difference in visible light transmittance at low temperature T_lum,lt_, and high temperature T_lum, ht_, the calculation formula is as follows.

(7) $$\Delta T_{lum}=T_{lum,lt}-T_{lum,\;ht}$$

The critical transition temperature (Tc) is defined as the average of the transition temperature of T_H_ in the heating process and the transition temperature of T_L_, in the cooling process. It can be expressed as follows. The critical transition temperature (Tc) is defined as the average of the T_H_ transition temperature during heating and the T_L_ transition temperature during cooling. It can be expressed as follows.

(8) $$Tc=\frac{T_{H}+T_{L}}{2}$$

During thermal cycling experiments, a resistive heating stage was used to control the sample temperature. In contrast, an infrared thermal imager was used to evaluate the surface temperature of the thin films. This setup allowed for in situ optical measurements at various temperatures. The Fourier Transform Infrared Spectroscopy (FTIR) (Nicolet iS5, Thermo Scientific, Waltham, MA, USA) was used to measure transmission spectra in the infrared range. The measurement range for this study was from 7800 cm^−1^ to 350 cm^−1^.

## 3. Experimental Results

### 3.1. Transmission Spectra of Different Thin Film Structures

**Figure 3:**
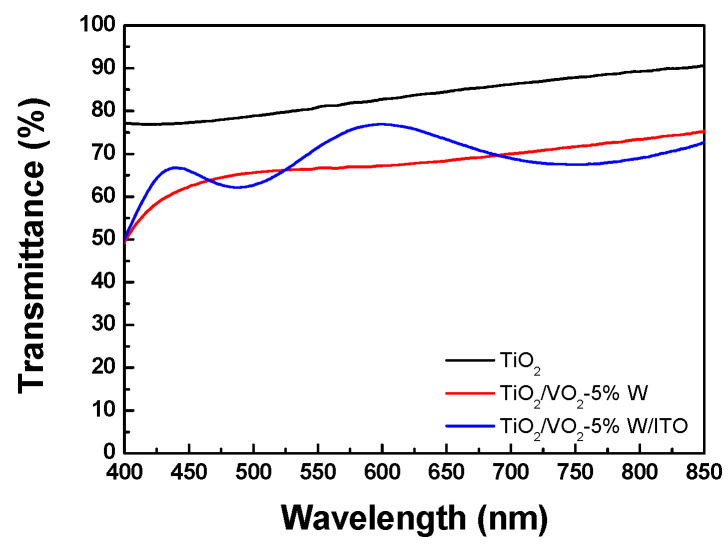
Transmission spectra of TiO_2_, VO_2_-5%W/ TiO_2_, ITO/VO_2_-5%W/TiO_2_ thin films.

The visible light spectrometer was utilized to measure the optical transmittance of two distinct thin film structures: TiO_2_/B270, VO_2_-5%W/TiO_2_/B270 (bilayer), and ITO/VO_2_-5%W/TiO_2_/B270 (trilayer). When TiO_2_ is served as the high refractive index thin film, the average transmittance in the visible light wavelength range (400 nm–750 nm) reaches 83.6%. This value indicates that TiO_2_ offers superior transparency in the visible spectrum. Upon depositing the VO_2_-5%W layer, the transmission spectrum shifted downward, resulting in an average transmittance decrease to 67.9%. This reduction is attributed to the optical absorption and scattering effects introduced by the VO_2_-5%W layer. Such changes are anticipated since the VO_2_-based layer is designed to provide thermochromic functionality with switchable optical properties. After the final deposition of the ITO buffer layer, the average transmittance increased from 67.9% to 69.8%. The employment of TiO_2_ as the top layer not only enhanced the visible transmittance but also improved the crystalline growth of the VO_2_ film. Consequently, the luminous transmittance (T_lum_) of the TiO_2_/VO_2_-5%W/ITO trilayer structure was evaluated to be 69.8%, as shown in Figure 3.

**Figure 4:**
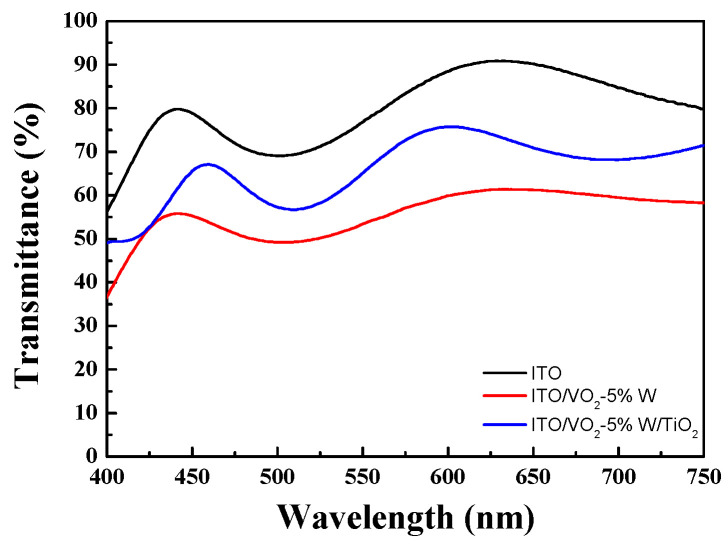
Transmission spectra of ITO, VO_2_-5%W/ITO, TiO_2_/VO_2_-5%W/ITO thin films.

The second type of asymmetric multilayer structure was created by reversing the stacking sequence to form a TiO_2_/VO_2_-5%W/ITO/B270 configuration. The transmission spectra of each layer arrangement were analyzed accordingly. Figure 4 illustrates the transmittance of the ITO/B270, VO_2_-5%W/ITO/B270 (bilayer), and TiO_2_/VO_2_-5%W/ITO/B270 (trilayer) structures. When ITO was applied as a single-layer film, the average transmittance in the visible wavelength range (400 nm–750 nm) reached 75.3%. This indicates that ITO has excellent transparency, making it a suitable base material for optical components. By utilizing the bilayer structure of VO_2_-5%W/ITO as a high-reflection coating, the addition of a third TiO_2_ layer increased the average visible transmittance from 56.4% to 66.1%. This 10.3% enhancement in transmittance shows that the TiO_2_/VO_2_-5%W/ITO structure significantly improves overall optical transmission. As a result, the luminous transmittance (T_lum_) of the TiO_2_/VO_2_-5%W/ITO trilayer structure was determined to be 66.1%, as depicted in Figure 4.

### 3.2. Transmission Spectra of Asymmetric Trilayer Thin Films During Heating and Cooling Processing

**Figure 5:**
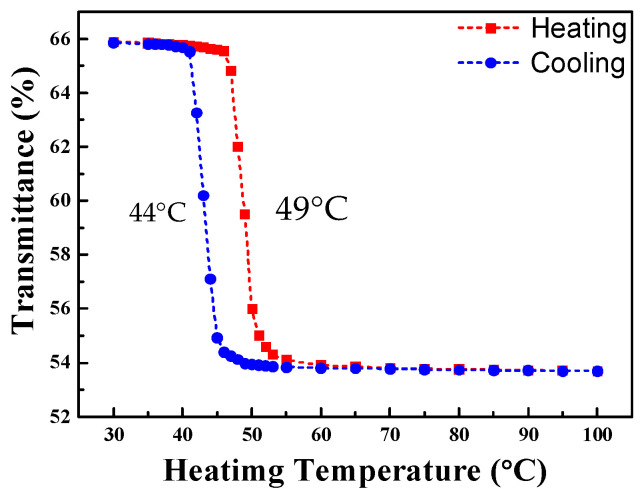
Transmission spectra of TiO_2_/VO_2_-5%W/ ITO/B270 during heating and cooling processing.

**Figure 6:**
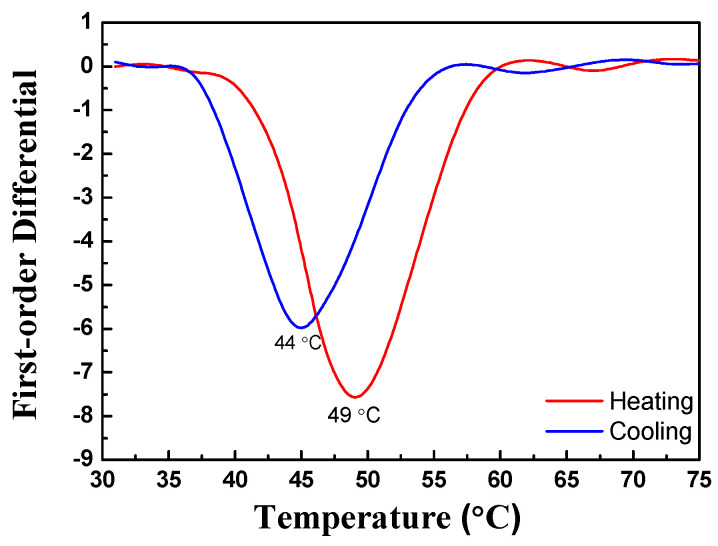
First-order differential curves of TiO_2_/VO_2_-5%W/ITO/B270 structure during heating and cooling processing.

**Figure 7:**
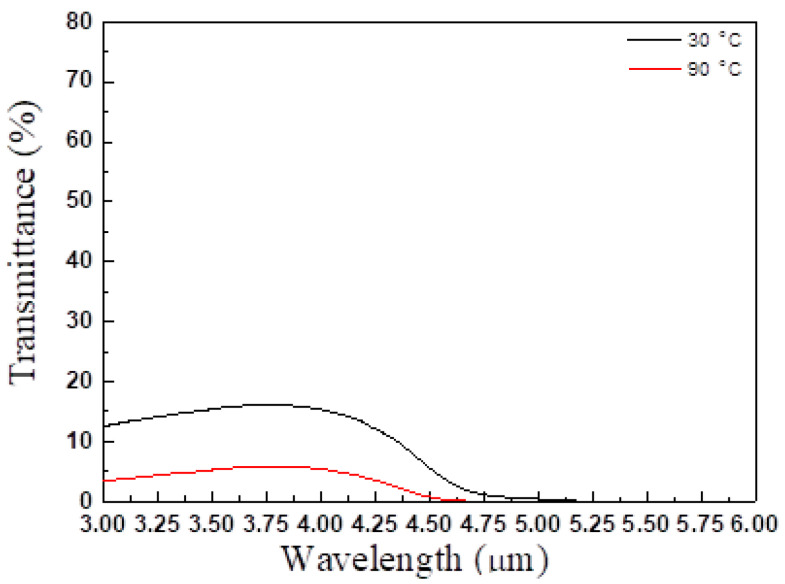
TiO_2_/VO_2_-5%W/ ITO/B270 spectra at 30 °Cand 90 °Cin the MIR band.

For the type I trilayer thin film structure (TiO_2_/VO_2_-5%W/ITO/B270), the transmission spectrum during the heating and cooling experiment is shown in Figure 5. The results indicate that the average transmittance began to decrease when the temperature reached approximately 47 °C. When the temperature further increased to 51 °C, the transmittance stabilized. From this behavior, the heating transition temperature (T_H_) was determined to be 49 °C. During this process, the average transmittance decreased from an initial 66.1% to 53.7%. In the cooling experiment, the transmittance began to recover when the temperature dropped to 46 °C, reaching 65.7% at 42 °C. Consequently, the cooling transition temperature (T_L_) was calculated to be 44 °C, yielding a critical phase transition temperature (T_C_) of 46.5 °C. The hysteresis width ΔT is defined as the absolute value of the difference between T_H_ and T_L_. Figure 5 shows that the deposited three-layer film exhibits a narrow hysteresis loop whose ΔT is 5 °C. By applying Equation (7), the luminous transmittance difference (ΔT_lum_) was calculated to be 12.4%. The temperature-dependent transmittance data were further analyzed using a first-order derivative method, as illustrated in Figure 6. Each of the first-order differential curves is fitted with Gaussian function using the peak fitting module. The temperature T_H_ = 49 °C and T_L_ = 44 °C represent the transition temperature upon heating and cooling, respectively. Figure 7 presents the mid-infrared (MIR) transmittance spectra. At room temperature, the average mid-infrared transmittance (T_MIR_) was 13.2%, decreasing to 5.2% when the sample was heated to 90 °C. The resulting transmittance modulation (ΔT_MIR_) was 8.0%.

**Figure 8:**
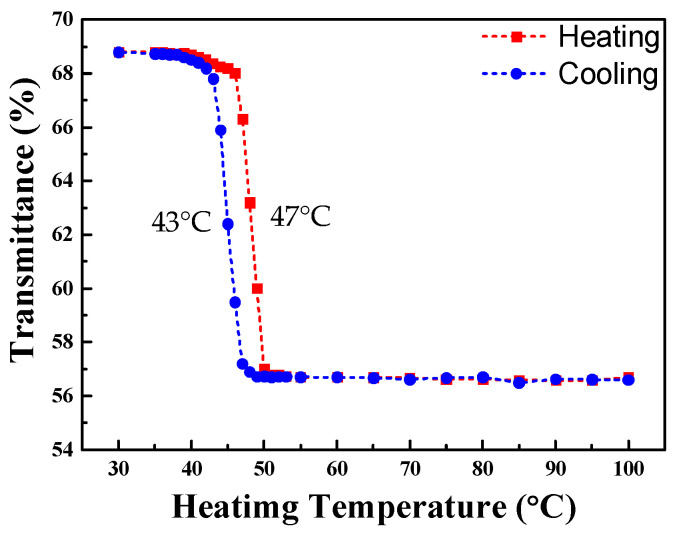
Average transmission spectra of ITO/VO_2_-5%W/TiO_2_/B270 in the visible light band at heating and cooling processes.

For the type II composite film (ITO/VO_2_-5%W/TiO_2_/B270), the transmission spectrum during the heating and cooling experiment is shown in Figure 8. The experimental results indicate that when the heating temperature reaches 45 °C, the average transmittance of the thin film decreases. As the temperature further increases to 50 °C, the transmittance stabilizes, from which the heating transition temperature (T_H_) is calculated to be 47 °C. During this process, the average transmittance decreases from the initial 69.8% to 56.5%. In the cooling experiment, it is observed that the average transmittance gradually recovers as the temperature drops to 45 °C, reaching 65.7% at 41 °C. From this, the cooling transition temperature (T_L_) is determined to be 43 °C, yielding a critical phase transition temperature (T_C_) of 45 °C. Using Equation (2), the luminous transmittance difference (ΔT_lum_) is calculated as 13.3%.

**Figure 9:**
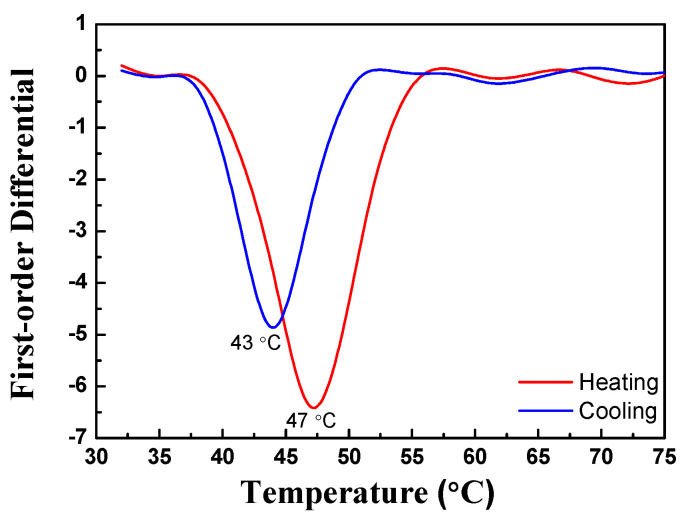
First-order differential curves of average transmittance of ITO/VO_2_-5%W/TiO_2_/B270 at different temperatures.

Figure 9 shows the first-order differential curves of average transmittance of ITO/VO_2_-5%W/TiO_2_/B270 at different temperatures. Each of the first-order differential curves is fitted with Gaussian function using the peak fitting module. As can be seen from Figure 9 that the type II trilayer film stack exhibits a narrow hysteresis loop. The hysteresis width ΔT is as small as 4 °C, which can be explained on the basis of the irregular shape particle and the large transversal grain size [42].

### 3.3. Raman Spectroscopy Analysis

A detailed analysis of the thin-film samples was conducted using Raman spectroscopy to investigate their molecular structure and chemical composition. Particular attention was given to the changes in Raman spectra under different temperature conditions, to understand the influence of temperature on the film’s composition. During the heating process, significant changes in molecular structure and chemical bonding may occur, which would be reflected in the Raman spectra. By measuring the Raman spectra at various temperatures, we observed changes in molecular vibrational modes, which allowed us to analyze the thermal stability and structural transitions of the film.

**Figure 10:**
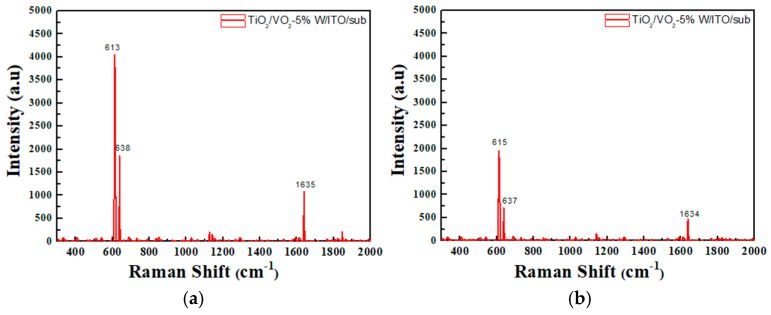
Raman spectra of ITO/VO_2_-5%W/TiO_2_ coated on a silicon wafer at different temperatures (**a**) 36 °C; (**b**) 90 °C.

**Figure 11:**
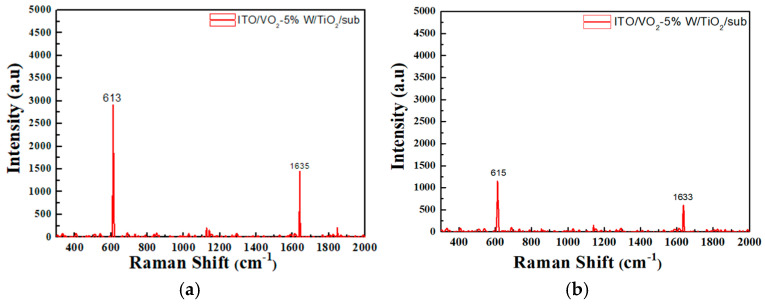
Raman spectra of TiO_2_/VO_2_-5%W/ITO coated on a silicon wafer at different temperatures (**a**) 36 °C; (**b**) 90 °C.

For instance, certain chemical bonds may break or new bonds may form at elevated temperatures, which can be detected through shifts in peak positions, variations in intensity, and changes in the shape of the Raman spectra. As shown in Figure 10 and Figure 11, the left column presents the surface temperatures of the films observed via thermal imaging. In contrast, the right column lists the observed shifts in VO_2_ Raman peaks during heating, from 613 cm^−1^ to 615 cm^−1^, accompanied by a decrease in intensity. This shift corresponds to a phase transition from the monoclinic (insulating) phase to the tetragonal (metallic) phase. Both types of composite films exhibit structural changes in their crystal lattices due to this phase transition, which consequently affect the vibrational modes observed in the Raman spectra. The Raman peak at 613 cm^−1^ typically corresponds to the lattice vibration of the monoclinic phase of VO_2_. As temperature increases, VO_2_ gradually transitions into the tetragonal phase, altering its vibrational characteristics and causing the peak to shift to 615 cm^−1^. The reduction in Raman peak intensity suggests a decrease in lattice order with increasing temperature. At higher temperatures, intensified lattice vibrations result in a weakening of the Raman scattering intensity. This observation is consistent with the literature [43], which reports a blue shift in Raman spectra upon heating of VO_2_ samples.

### 3.4. Residual Stress at Different Heating Temperatures

Residual stress plays a crucial role in determining the properties of thin films, affecting their mechanical stability, adhesion strength, optical properties, and phase transition behavior. Especially under different temperature conditions, the influence of residual stress and thermal stress on multilayer films is particularly significant. We understand the PTT changes in thermochromic films through measurements and data analysis. In VO_2_-based thin films, residual stress can alter lattice parameters, thereby changing the PTT. In addition, excessive tensile or compressive stress can lead to cracking, delamination, or decreased optical performance of thin film components.

In this work, the VO_2_-based trilayer thin films were simultaneously deposited onto B270 and H-K9L glass substrates. Prior to and after the deposition process, a Twyman–Green interferometer was employed to measure surface deformation. Interference patterns were captured using a CCD camera. By combining the FFT method with the phase unwrapping technique, the surface profile of the trilayer thin films was reconstructed to calculate the ROC. This value was then used in the Stoney equation to determine the residual stress in thin films. Two different VO_2_-based thin film structures, TiO_2_/VO_2_-5%W/ITO/B270 and ITO/VO_2_-5%W/TiO_2_/B270, were analyzed. Measurements were performed at 10 °C intervals from room temperature (30 °C) up to 120 °C. By fitting the slope of the residual stress–temperature plot obtained from each substrate, and incorporating the corresponding substrate parameters, as listed in Table 1. The CTE and biaxial modulus were calculated by analyzing the slopes of the residual stress–temperature curves of the two glass substrates.

**Figure 12:**
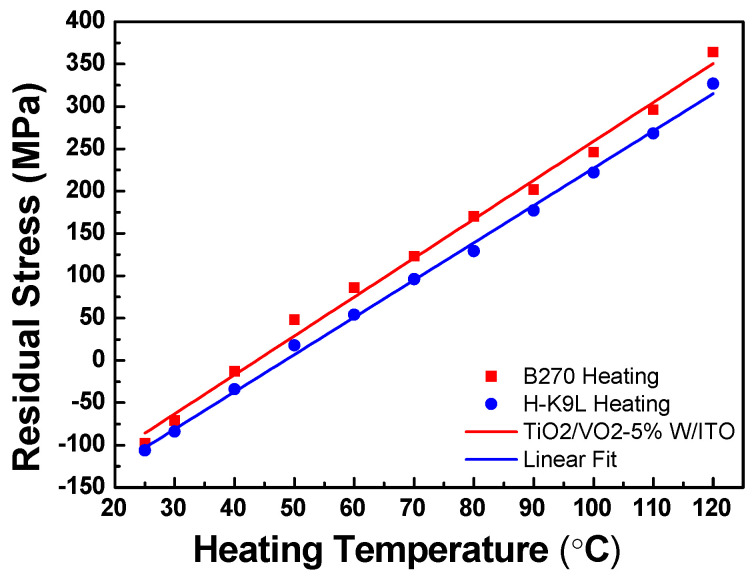
Linear fitting plot of residual stress of TiO_2_/VO_2_-5%W/ ITO films deposited on B270 and H-K9L glass substrates as a function of heating temperature.

**Figure 13:**
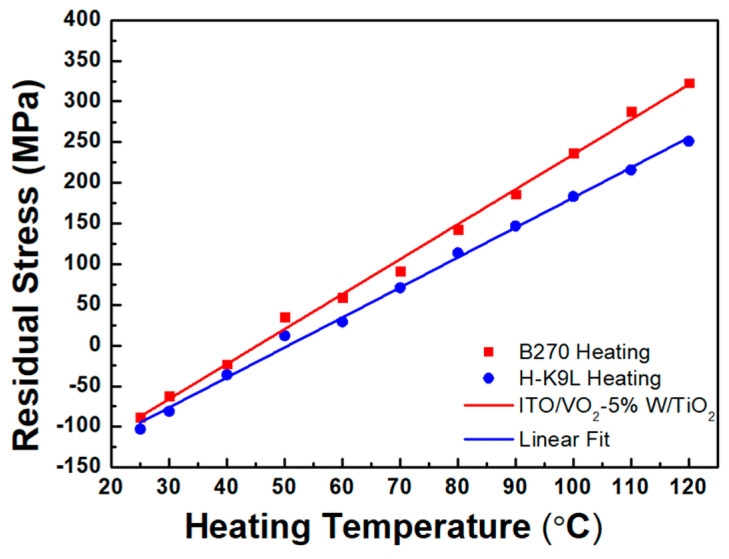
Linear fitting plot of residual stress of ITO/VO_2_-5%W/TiO_2_ films deposited on B270 and H-K9L glass substrates as a function of heating temperature.

Thermal stress analysis was conducted on TiO_2_/VO_2_-5%W/ITO/B270 trilayer thin films deposited on different glass substrates. After the three-layer thin film deposition was completed, the thermal stress was measured at different heating temperatures. The measurement results obtained from the B270 and H-K9L glass substrates are shown in Figure 12. During the heating process, the residual stress value increased progressively from an initial compressive stress of −98 MPa. At approximately 47 °C, the residual stress transitioned from a compressive state to a tensile state. The slopes of stress–temperature curves of the B270 and H-K9L substrates were obtained by using linear fitting. Figure 12 shows that the fitting slopes for TiO_2_/VO_2_-5%W/ITO trilayer thin film deposited on the B270 and H-K9L glass substrates are 4.29 and 3.68, respectively. Substituting the parameters into Equations (4) and (5) for both glass substrates yields a CTE and biaxial modulus of 5.37 × 10^−6^ °C^−1^ and 295.7 GPa. Figure 13 shows that the fitting slopes for ITO/VO_2_-5%W/TiO_2_ trilayer on the B270 and H-K9L glass substrates are 4.59 and 4.42, respectively. Substituting the parameters for both substrates yields a CTE of 6.65 × 10^−6^ °C^−1^ and a biaxial modulus of 745.0 GPa, respectively. The mechanical properties of the trilayer film structures, characterized by their thermal expansion coefficients and biaxial moduli, further support the role of multilayer coupling. The significantly higher biaxial modulus observed in the ITO/VO_2_-5%W/TiO_2_ structure (745.0 GPa) compared to TiO_2_/VO_2_-5%W/ITO (295.7 GPa) indicates a stronger constraint on the lattice deformation, which enhances residual stress transfer across interfaces.

Thermal stress analysis of ITO/VO_2_-5%W/TiO_2_/B270 thin films was also conducted by dual-substrate method. The multilayer thin films were deposited on B270 and H-K9L glass substrates, followed by thermal stress measurements. During the heating process, the residual stress increased progressively from an initial compressive stress of −88 MPa. At approximately 50 °C, the residual stress transitioned from compressive to tensile. The comparison of the measurement results of the two trilayer thin film structures is shown in Table 2. According to the Raman spectroscopy analysis, in all three-layer thin film configurations, the peak wavenumber of the three-layer thin film shifts from 613 cm^−1^ to 615 cm^−1^ upon heating, while the peak intensity decreases. This blue shift and intensity decrease may be attributed to a reduction in moisture content, leading to a denser film structure, as well as the phase transition of W-doped vanadium oxide from a low-temperature insulating phase to a high-temperature metallic phase. In terms of optical properties, the trilayer thin film design successfully enhanced the visible light transmittance (T_lum_) from 63.2% for a single-layer VO_2_ to 69.8%. In comparison, the mid-infrared transmittance (T_Mir_) decreased significantly from 35.52% to 16.8%. Heating experiments showed that the critical phase transition temperature (T_c_) of the ITO/VO_2_-5%W/TiO_2_/B270 structure was significantly reduced from 68 °C to 45 °C, demonstrating the effectiveness of the multilayer design in improving the phase transition characteristics of VO_2_-based thin films. To verify the practical application value of the proposed thin films, we integrated the optimized three-layer structure into the smart window prototype. Based on the measured transition temperature (~45 °C), high visible light transmittance (~70%), and significant infrared modulation, this trilayer thin film will be applied towards energy-saving coatings.

The above experimental measurements show that in ion-assisted deposition, the additional kinetic energy provided by ion bombardment enhances atomic mobility and promotes the formation of non-equilibrium defects. In the asymmetric three-layer structure, this internal stress is further amplified and redistributed due to the elastic and thermal mismatch between adjacent layers. The coupling between dopant-induced lattice distortion and multilayer stress evolution leads to changes in the VO_2_ free energy distribution, thereby reducing the PTT. In addition, the enhanced interfacial bonding strength observed in the multilayer thin film structure is related to the increase in cohesive energy at the interface, as shown by the theoretical model study [23]. Stronger interfacial bonding improves mechanical stability, reduces defect density, and contributes to the reduction in observed residual stress fluctuations. The consistency between experimental observations and theoretical predictions further validates the effectiveness of the proposed VO_2_-based trilayer thin film design.

## 4. Conclusions

To reduce the critical phase transition temperature of W-doped vanadium dioxide thin films for applications like smart windows, this work proposes VO_2_-based trilayer structures. By designing and fabricating two types of trilayer thin film structures, the optical performance of VO_2_-based films was significantly enhanced, and their temperature-dependent residual stresses were better understood. Notably, the critical phase transition temperature of the ITO/VO_2_-5%W/TiO_2_ trilayer thin films was successfully reduced to 45 °C. The results show the effectiveness of the multilayer design in improving the phase transition characteristics of VO_2_-based thin films. For the optical properties, the trilayer thin film design successfully enhanced the visible light transmittance (T_lum_) from 63.2% for a single-layer VO_2_ film to 69.8%. In comparison, the mid-infrared transmittance (T_MIR_) decreased significantly from 35.52% to 16.8%. For the temperature-dependent residual stress measurements, the residual stress of the trilayer thin films during heating experiments revealed that the residual stress shifted from compressive to tensile in the temperature range of 40 °C to 50 °C. Based on double substrate and curve fitting methods, the CTE and biaxial modulus of the TiO_2_/VO_2_-5%W/ITO three-layer film structure were 5.37 × 10^−6^ °C^−1^ and 295.7 GPa, respectively. In contrast, the CTE and biaxial modulus of the ITO/VO_2_-5%W/TiO_2_ three-layer film structure were 6.65 × 10^−6^ °C^−1^ and 745.0 GPa, respectively. Although these improvements substantially enhance the film’s performance, further development is still needed to meet the practical demands of smart window applications. As ambient temperatures continue to rise, lowering the PTT to near 25 °C would improve the performance of smart windows in real-world environments. In future work, we will focus on integrating these thin films into functional components, continuously optimizing the thin film layer structure to reduce the PTT to room temperature, and evaluating their long-term durability and large-area scalability.

## Figures and Tables

**Table 1:** Physical parameters of different glass substrates.

Glass Substrate	B270	H-K9L
CTE (°C^−1^)	8.2 × 10^−6^	7.6 × 10^−6^
Young’s modulus (GPa)	71.5	79
Poisson ratio	0.219	0.214
Thickness (mm)	1.5	1.5

**Table 2:** Comparison of the measurement results of TiO_2_/VO_2_-5%W/ITO/B270 and ITO/VO_2_-5%W/TiO_2_/B270 multilayer films.

Compared Items	TiO_2_/VO_2_-5%W /ITO/B270	ITO/VO_2_-5%W /TiO_2_/B270
Visible-light band transmittance	66.1%	69.8%
Mid-Infrared band transmittance	13.2%	16.8%
Critical phase transition temperature	46.5 °C	45 °C
Residual stress for B270 glass (from 25 °C to 120 °C)	−88 to 323 MPa	−98 to 364 MPa
Residual stress for H-K9LB270 glass (from 25 °C to 120 °C)	−101 to 251 MPa	−106 to 327 MPa
CTE	5.37 × 10^−6^ °C^−1^	6.65 × 10^−6^ °C^−1^
Biaxial modulus	295.7 GPa	745.0 GPa
Raman peak wavenumber	613 cm^−1^	613 cm^−1^

## Data Availability

The original contributions presented in this study are included in the article. Further inquiries can be directed to the corresponding author.
